# Design of a Network Optimization Platform for the Multivehicle Transportation of Hazardous Materials

**DOI:** 10.3390/ijerph17031104

**Published:** 2020-02-10

**Authors:** Sheng Dong, Jibiao Zhou, Changxi Ma

**Affiliations:** 1 School of Civil and Transportation Engineering, Ningbo University of Technology, Ningbo 315211, China; dongsheng@nbut.edu.cn; 2 College of Transportation Engineering, Tongji University, Shanghai 200082, China; 3 Intelligent Transport System (ITS) R & D Center, Shanghai Urban Construction Design and Research Institute (Group) Co., Ltd., Shanghai 200082, China; 4 School of Traffic and Transportation, Lanzhou Jiaotong University, Lanzhou 730070, China

**Keywords:** hazardous materials, transportation network, optimization platform, design, safety

## Abstract

With economic development, the volume of hazardous materials is increasing, and the potential risks to human beings and the natural environment are expanding. Road transportation has become the main mode of transportation for hazardous materials. Because of the specific characteristics of hazardous materials, if an accident occurs in the transportation process, it often causes mass casualties, serious property and socioeconomic damage, and damage to the ecological environment. Hence, transportation is an important part of the life cycle of hazardous materials. This paper designs an optimization platform for multidestination, multiterminal, and multivehicle networks that transport hazardous materials. The logistics module in TransCAD software is used to construct this platform. By identifying the effective transportation routes considering the transportation risk, sensitive target population, and transportation time of each road section, the entropy method can be used to fuse and obtain the comprehensive impedance value of each road section. Finally, the optimal transportation network of hazardous materials was obtained by the transportation network optimization algorithm in TransCAD. The platform can display the optimal transport program with data windows, text, and maps. The research results provide a reference for relevant departments to scientifically manage the transport of hazardous materials.

## 1. Introduction

Hazardous materials are substances with explosive, flammable, poisonous, corrosive, and other characteristics [1,2]. These materials require special protection during transportation, handling, and storage, because they can cause personal injury, property damage, and environmental pollution. According to statistics, the hazardous materials that need to be transported throughout the world each year amount to more than 4 billion tons, and the amount of hazardous materials in China alone exceeds 400 million tons per year [3]. Approximately, 95% of hazardous materials are moved via long-distance transportation, and approximately 80% are transported via roads. Many flammables, explosive, poisonous, and corrosive hazardous materials are transported along road networks, thus representing potential “time bombs” that threaten the lives and property of people that live near the transportation route. Although the rate of transportation accidents involving hazardous materials on roads is relatively low [4,5], this issue has attracted attention from all sectors of society, due to the large transportation volume, numerous risk factors, and complicated and variable transportation environments [6].

**Figure 1:**
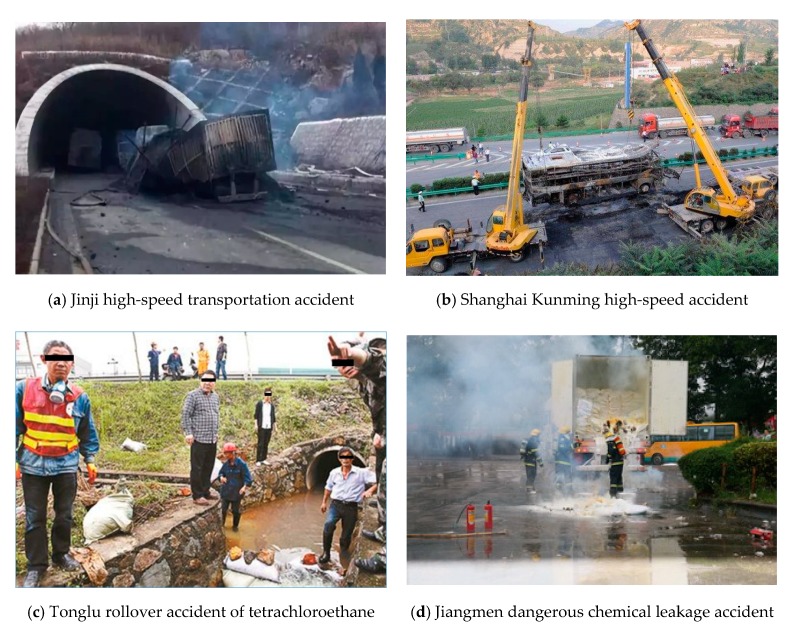
Hazardous materials transportation accidents.

In recent years, hazardous material transportation accidents in China have occasionally occurred [7,8]. From 2008, there have been many road transportation safety accidents involving extremely hazardous materials around the country. Notably, on 26 August 2012, the Baomao high-speed methanol transportation explosion accident occurred. On 1 March and 19 July 2014, the Jinji high-speed methanol transportation tunnel explosion accident and Shanghai Kunming high-speed ethanol transportation fire accident, which caused more than 30 deaths, occurred. Additionally, on 22 November 2016, the leakage accident of dangerous compound transportation in Jiangmen resulted in the evacuation of several blocks. The scenes of the above accidents are shown in Figure 1.

The safety management of the road transportation of hazardous materials has attracted extensive attention from society [6,7,8]. Hazardous materials can not only cause considerable casualties and property losses, but also cause serious damage to the natural environment and ecological systems. If sensitive areas in the natural environment are polluted by hazardous chemicals, the ecosystem may be seriously damaged and be difficult to repair in a short time, resulting in a dramatic loss of natural resources. On 18 May 2014, a rollover accident of a tetrachloroethane tank car in Tonglu, Zhejiang Province, caused the serious pollution of the Fuchun River, which led to the shutdown of Fuyang City, Zhejiang Province, located downstream. Serious fire and explosion accidents in the dangerous goods warehouse of the “August 12” Ruihai company in Tianjin Port, on 12 August, resulted in more than 100 kinds of residual chemicals and secondary pollutants being emitted, which resulted in varying degrees of pollution to the atmospheric environment, water environment, and soil environment in various areas.

To avoid similar accidents, improving the professional quality and regulatory awareness of hazardous chemical transportation practitioners is important. This approach can reduce the accident rates and number of casualties associated with hazardous material transportation, and the safety risks of the route can be evaluated to select the route with the highest safety level and avoid densely populated areas. Therefore, it is of great practical significance to reasonably plan the road transportation routes of hazardous chemicals transport to reduce the public safety risks along the routes and improve the level of natural environmental protection. Under such circumstances, studies of the optimal design of hazardous material transportation networks can help identify safe and efficient transportation networks for vehicles. This approach will help improve the safety management levels of government regulatory agencies and production and business units and prevent and control the occurrence of road transportation accidents involving hazardous materials.

Many experts and scholars have conducted research on the optimal design of hazardous material transportation networks. Batta and Chiu [9] defined and studied the risk of hazardous material transportation. They divided the risks of hazardous material transportation into two parts: the risk associated with transportation road sections and the risk at transportation nodes. The sum of the risks was considered the overall transportation risk between two points, and the length of the road section and the population density were comprehensively considered. Karkazis and Boffey [10] established a risk transportation route optimization model by using lowest-risk and lowest-cost objective functions. Zografos and Androutsopoulos [11] treated the vehicle routing problem involving hazardous material transportation as a biobjective vehicle routing problem with time window constraints and proposed a heuristic algorithm to solve the problem [11,12]. Meng et al. [13] established a time-constrained multiobjective hazardous material transportation route optimization model and adopted a dynamic programming method to solve the problem. Finally, a case study was conducted. Das et al. [14] studied the transportation route problem of hazardous waste in a transportation network with capacity constraints. Using a multiobjective algorithm, they solved the network transportation problem with multiple destinations and terminals and found the nondominated solutions. Pradhananga [15] considered the total transportation time and the total transportation risk minimum as the optimization objectives, established a biobjective risk transportation route optimization model with time windows, and designed a heuristic algorithm to find the Pareto optimal solutions. Ma et al. [16] established a hazardous material route selection model under time-varying conditions. Liu et al. [17] applied a multilevel fuzzy comprehensive evaluation method to optimize hazardous material transportation routes and established a fuzzy comprehensive evaluation model. Kara et al. [18] studied actual accident cases and the optimization of multiobjective hazardous material transportation routes. Ma et al. [19,20,21,22] analyzed transportation route selection for hazardous materials in a stable environment and an uncertain environment and proposed a multiobjective route planning model for hazardous material transportation in a stable environment. In the uncertain environment, the multiobjective path probability-constrained model and the multiobjective path probability-dependent model for hazardous material transportation were proposed. Ghaderi and Robert [23] proposed an integrated location and routing approach for transporting hazardous materials in a bimodal transportation network. López-Ramos et al. [24] presented an integrated model and specialized local search for the road network pricing and the design of hazmat vehicles. In addition, Feng Chen et al. [25,26,27] analyzed the accidents involving drivers that transported goods under bad driving conditions. Zhou et al. [28,29,30] explored the interactions between safety risk of urban large-scale public spaces and its influencing factors. The full Bayesian random parameters multivariate Tobit model was used by Guo et al. [31,32] to evaluate the safety impacts and modeled correlation and heterogeneity in crash rates by collision types. Zhao et al. [33] presented an unsupervised two-phase framework for inferring multiple trip purposes (i.e., loading, unloading, in-yard, and other stops) based on the passive global positioning system (GPS) data during the hazardous materials transportation process. Ma et al. [34] reviewed the optimization of hazardous materials transportation from transportation risk, route optimization, and fleet scheduling.

Obviously, there have been numerous achievements in the current optimization design of hazardous material transportation networks, but the network design solutions obtained from these studies have been poorly visualized. Because TransCAD [35,36] is a software program that combines geographic information systems, transportation planning models, and logistics modules, it performs well in map visualization. This paper attempts to develop a software platform to design and optimize multidestination, multiterminal, and multivehicle transportation networks for hazardous materials based on TransCAD.

## 2. Basic Data System of Optimization Platform of the Hazardous Materials Transportation Network

### 2.1. Basic Data Stratification

TransCAD software hierarchically stores and manages information with geographic features. That is, different geographic information is stored in different layers, and geographic objects with the same characteristics are stored in a geographic layer. Moreover, each layer requires the support of the database table, and a layer corresponds to a database table. An object in a layer corresponds to a record in the database table. By overlaying multiple layers, area information can be intuitively studied. This article divides the entire network into three geographic layers.

(1) Road network layer

The road sections are represented by a line geographic file. All the road sections form a road network layer. The role of this layer is to store the road network, and it is linked to the road-related geographic data.

(2) Customer point layer

The customer point layer is a geographical layer for storing customer points, and it is associated with all geographic information data related to logistical facilities and service objects.

(3) Facility point layer

The facility point layer is a geographic layer used to store logistical facility points. This layer is associated with all the data related to logistics facility points. The three geographic layers can be superimposed to visualize the layout of the road network and the distributions of facilities and customers.

### 2.2. Basic Data Composition

(1) Road network data

The information attributes of the road network include the length of each road section, the population on both sides of a route, the vehicle speed of a road section, and the travel time along a road section. In TransCAD, the elements of the transportation network are nodes and lines. Nodes are used to represent feature points, such as traffic hubs and road intersections, and lines represent road sections.

Length: The length of a road section in TransCAD refers to the route length between two road network nodes. TransCAD can calculate the length of each road section and automatically generate a road network property table.

Speed: The road section speed refers to the vehicle speed between two nodes of the road network.

Time: The road section travel time refers to the time spent by vehicles using the route to travel between two nodes in the road network. The unit is min. This value can be calculated using the formula Time = Length/Speed in the TransCAD data window.

(2) Facility data

The facility data mainly include the facility ID, facility name, facility service time, facility location, and node ID. The Facility ID is an inherent attribute of the facility layer, which distinguishes different facilities by using numbers. The facility name (NAME), denoted by “F*”, * is a number, or directly uses a Chinese name. The facility location will be given directly and expressed in longitude and latitude coordinates in TransCAD. After importing the AutoCAD road map into TransCAD, latitude and longitude registration will be performed according to the selected area. The facility service time refers to the time at which the facility provides a delivery service. This time definition requires two boundary values, namely the service start time of the facility (OPEN TIME) and the stop service time of the facility (CLOSE TIME). This time interval is the normal service time interval of the facility, and facilities will provide logistical and distribution services to customers. Outside this time interval, the facility will stop providing services to customers. This paper sets all facility service time intervals to 0000−2400 to provide services 24 h a day. The time format is military format, i.e., 1800 is 6:00 PM. The facility node ID (NODE ID) is the ID nearest to the facility point in the road network, and this ID is used to relate the facility point layer and the road network layer. The data table of the facility point layer is shown in Table 1.

(3) Customer data

These data mainly include the customer ID, customer name, customer location, customer demand, and customer business hours. The customer ID is an inherent attribute of the customer’s point layer and is distinguished by numbers. The customer name (NAME) is denoted by “C*”. The customer location is given directly and is expressed in latitude and longitude coordinates. The customer business hours refer to the time when the customer accepts delivery services. This variable requires two boundary values, namely the customer’s opening hours (OPEN TIME) and the customer’s closing hours (CLOSE TIME). This time period establishes the normal business hour interval for each customer. During this time interval, customers will receive the logistical and distribution services provided by the facilities. The fixed service time (min) refers to the time between when a transportation vehicle arrives at a customer location and must stay for a given amount of time, such as to perform checks or inspections; this time is denoted as the FIXED SERVICE TIME in the attribute field. The unit unloading time (min) refers to the average time required by the unloading personnel at the customer location to unload one unit of goods, which is denoted by TIME PER UNIT in the attribute field. Customer demand volume (DEMAND) refers to the customer’s demand for goods. For different customers, the demand is not the same, so the degree of importance is also different. The customer demand in this paper does not set the unit. It only represents customer demand weight. The node ID (NODE ID) refers to the node ID nearest to the customer point in the road network. This field is used to relate the customer point layer to the road network layer. The data table of the customer point layer is shown in Table 2.

(4) Vehicle data

The vehicle data mainly include the vehicle type, number of vehicles at various facility points, vehicle capacity, and vehicle operating costs. Depot ID refers to the ID of the facility at which the vehicle is located. This attribute is used to link vehicle information and facility point layer information. The vehicles at facility points are divided into three types, namely, A, B, and C, according to the vehicle capacity, as shown in Table 3. The vehicle capacity indicates the maximum load of the vehicle, that is, the upper limit of the vehicle carrying capacity. As with the customer demand, the vehicle capacity does not have actual units in this study. The number of vehicles at each facility point (Num-Vehs) is the number of vehicle types owned by each facility. The vehicle cost includes the fixed cost of each type of vehicle purchased or leased by a facility and variable operating cost of each vehicle. The vehicle data are shown in Table 3.

## 3. Key Elements of the Multidestination, Multiterminal, and Multivehicle Transportation Network Optimization for Hazardous Materials

### 3.1. The Screening of Effective Road Sections

The risk values of different road sections differ greatly, depending on whether or not there are key protection measures implemented for events, such as hazardous material spills or explosions. If the affected area contains key protected places (water sources, chemical plants, power facilities, military control areas, etc.), then the road section is considered an invalid road section, and hazardous materials vehicles are not allowed to drive on these road sections.

The optimization platform treats the road sections that hazardous material vehicles use for travel as the elements and the radius of influence of hazardous material leakage or explosion events as the buffer zone. The attribute screening function in TransCAD is used to determine whether the buffer zone contains key protection areas; if so, the road section is deemed an invalid road section, and the road section impedance is set to infinity. Conversely, if no protection areas are found, the section is considered an effective road section, and the algorithm is used to generate an optimal transportation path along effective road sections.

### 3.2. Key Parameters for Obtaining the Optimal Transport Network Solution

The hazardous material transportation risk, the number of sensitive target populations, and the transportation time are selected as the attribute parameters of the effective road section. The calibration method of each parameter is as follows.

(1) Transportation risk

To consider the road section transportation risks of hazardous materials, domestic and foreign scholars have conducted multifaceted measurements in terms of the environmental impact, road conditions, driver factors, and uncertainty factors. For ease of operation, the system in this study uses a traditional transportation risk definition model to quantitatively describe the road section transportation risks.

(1) $$R_{ij}^{k}=p_{ij}^{k}\cdot M^{k}$$

where $R_{ij}^{k}$ is the risk value of the hazardous materials k passing through the road section ij; $p_{ij}^{k}$ is the probability of an accident where hazardous materials k pass through the road section ij; and $M^{k}$ is the loss caused by an accident with hazardous materials k.

(2) Sensitive target population

The buffer analysis in TransCAD has powerful functions involving spatial map information retrieval and comprehensive information processing. These functions can accurately measure and visually display the population distributions around the road sections of hazardous material transportation. Taking the hazardous material transportation road sections as the element, the radius of influence of hazardous material spills or explosions can be used as the buffer zone, and the total population and number of coverage facilities (e.g., schools, hospitals, hotels, and attractions) can be used to determine the sensitive target population within a given buffer zone.

(2) $$Q_{ij}^{k}=\sum m_{r}\cdot pop_{r}$$

where $Q_{ij}^{k}$ is the sensitive target population of hazardous materials k passing through road segment ij; $m_{r}$ is the total number of places r in the buffer zone; and $pop_{r}$ is the population of the place r.

(3) Transportation time

The transportation time can be obtained according to the historical time records returned by the GPSs installed on hazardous material transportation vehicles. In the vehicle environment, the traffic information centre collects road network and the vehicle data through the road infrastructure. The relevant data are then processed to obtain traffic flow data for each monitoring point and road section, and transportation time can be obtained by the BPR function that is currently used by the US Highway Bureau.

### 3.3. Optimal Transportation Network Solution Process

**Figure 2:**
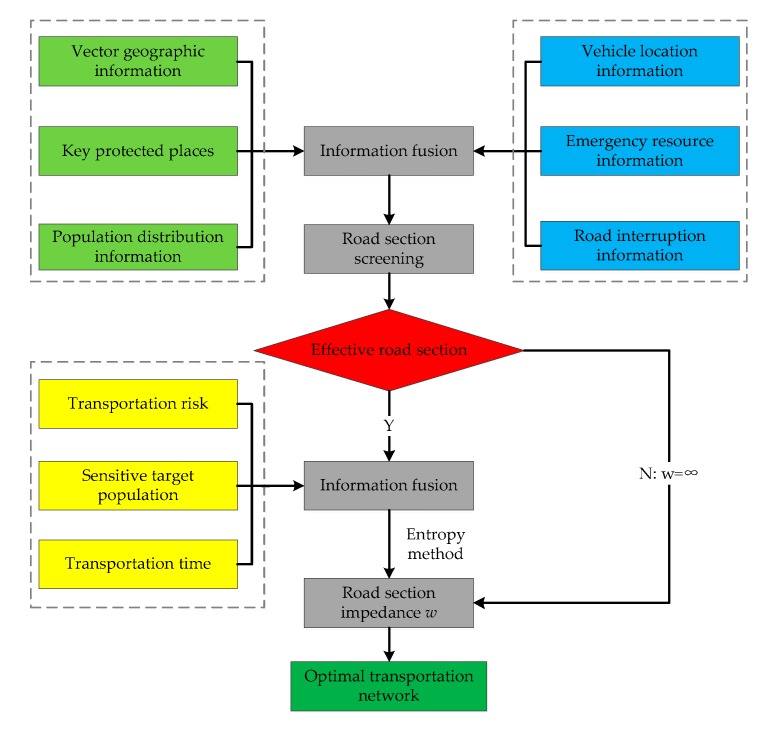
Algorithm flow chart for solving hazardous material optimal transportation network.

Based on the screening of effective transportation sections and the transportation risk, sensitive target populations, and transportation time, we use the entropy method to obtain the integrated impedance value for each road section. Finally, the multidestination, multiterminal, and multivehicle transportation network optimization algorithm in TransCAD can be used to obtain the optimal transport network of hazardous materials. The algorithm flow chart is shown in Figure 2.

## 4. Case Study

Large-scale hazardous material distribution centres are located in Donggang and Xigu, and these centres are responsible for transporting hazardous materials to 26 customers in the surrounding cities and counties. The demand point layer attribute data is shown in Table 4.

**Figure 3:**
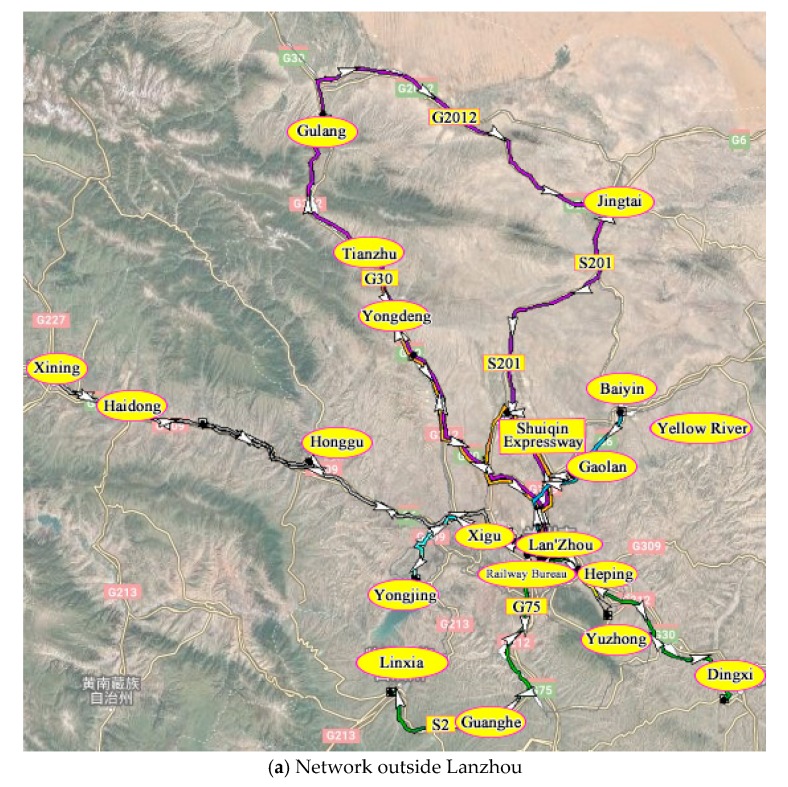

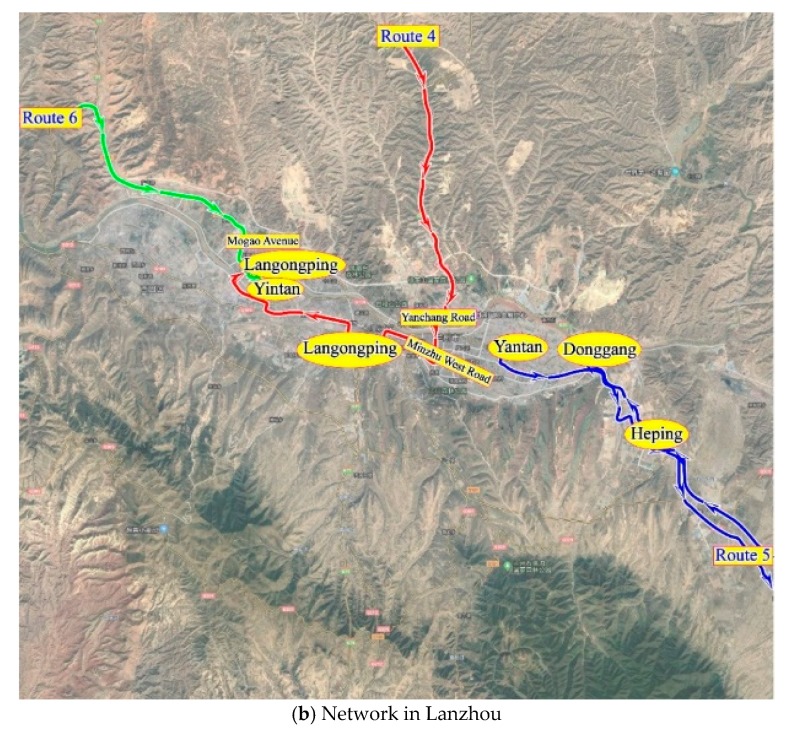
Hazardous material optimal transportation networks generated by the platform.

As described in Section 3 of this study, the detailed steps are, first, we screened the sections of the area involved in the transportation of hazardous materials, and the sections that were not suitable for the transportation of hazardous materials were removed. Second, the factors related to the transportation risk, transportation time, and sensitive target population were considered comprehensively, and then, the entropy method was used to calculate and obtain the comprehensive impedance value of each road section. Finally, the logistics module in TransCAD software was applied to calculate optimal transportation plan for the hazardous materials. The planned route direction was generated in the road network map. Different routes were indicated by different colors, and arrows indicate the travel direction, as shown in Figure 3. The specific path plan is shown in Table 5.

With the optimal platform, the specific transportation route and departure time of each vehicle transporting hazardous materials from each distribution centre can be obtained, and the specific driving route plan can be intuitively and clearly displayed on a map.

If we optimize the route of dangerous goods transportation vehicles according to the traditional method, which only considers the shortest driving time of vehicles and not the transportation risk and sensitive target population, the route of hazardous materials transportation can be obtained, as shown in Table 6.

From Table 5, plan A comprehensively considered transportation risk, transportation time, and sensitive target population. Compared with plan B, the transportation route experienced in plan A avoided the densely populated area, with less population along the transportation route, less transportation risk, and less increase in transportation time. In the process of transportation of hazardous materials, the most important thing was to ensure safety, and the short transportation time was only considered for the second step.

Although this optimal plan comprehensively considers the transportation risks, transportation time, and sensitive target populations, and it effectively avoids densely populated areas, there are fewer sensitive target populations along the selected transportation routes, the transportation risks are low, and the total transportation time is short. These results provide a valuable reference value for hazardous material transportation companies.

## 5. Conclusions

With the continuous socioeconomic development, the transportation volumes of various hazardous materials will continue to increase. The risk of hazardous material transportation accidents will also increase. Hazardous material accidents are different from ordinary traffic accidents and they are often sudden and destructive with a social impact. To reduce the accident rates of hazardous material transportation and scientifically guide first-line hazardous material enterprises in the transport of hazardous materials, it is necessary to optimize and design hazardous material transportation networks. This paper has designed an optimization platform for hazardous material transportation networks.

TransCAD is used to combine a variety of information involved in the optimization of hazardous material transportation networks and identify effective road sections suitable for hazardous material transportation. Then, the road transportation risk, sensitive target population, and transportation time are selected as optimization parameters. A hybrid algorithm is designed to solve the hazardous material optimal transportation problem for a given network. The optimization platform developed in this paper can rapidly generate and visually display the optimal transportation network. The results of repeated tests show that the optimization platform is stable in operation and can be used as an auxiliary decision-making system by the relevant management departments. Thus, this platform provides a certain reference value for improving the existing hazardous material transportation management systems.

The optimization platform uses an artificial vectorized map, and the production cost of this map is relatively large. In the future, the Baidu map API or the Gaode map API could be used to access the real-time electronic map. At the same time, an online version of the system could be released to further optimize the platform.

## Figures and Tables

**Table 1:** Facility point data type table.

Field Name	Data Type	Meaning
ID	Integer	Identify unique facilities
NAME	Character	Facility name
Longitude	Integer	Longitude coordinates of facility location
Latitude	Integer	Latitude coordinates of facility location
OPEN TIME	Integer	The earliest time of vehicle delivery
CLOSE TIME	Integer	The latest time the vehicle returns
NODE_ID	Integer	The node ID nearest to the facility point in the road network

**Table 2:** Customer point layer data type table.

Field Name	Data Type	Meaning
ID	Integer	Identify unique customers
NAME	Character	Customer name
Longitude	Integer	Longitude coordinates of customer location
Latitude	Integer	Latitude coordinates of customer location
OPEN TIME	Integer	The earliest time the customer can accept the service
CLOSE TIME	Integer	The latest time the customer can accept the service
FIXED SERVICE TIME	Integer	Fixed service time
TIME PER UNIT	Integer	Unit unloading time
DEMAND	Integer	Customer demand volume
NODE_ID	Integer	The node ID nearest to the customer point in the road network

**Table 3:** Vehicle classification and type.

Vehicle Type	A	B	C	
Vehicle capacity (t)	12	16	20	
Field name	Data type	Meaning		
Depot ID	Integer	Depot ID		
Capacity	Float	Vehicle load volume		
Type	Integer	Vehicle type		
Number of vehicles	Integer	Total number of vehicles at facility point		
Cost	Float	The fixed cost of each type vehicle		

**Table 4:** Demand point layer attribute data.

ID	Relative Longitude	Relative Latitude	Demand Point Name	Open Time	Close Time	Fixed Service Time (min)	Demand (t)
1	111832980	10401159	Mogao Dadao Intersection	13:00	18:00	20	1.8
2	111850054	10373911	Yintan	14:00	19:00	20	2.4
3	112017194	10337563	Yantan	8:00	12:00	30	3.5
4	111973124	10297599	Lanzhou Railway Bureau	8:00	16:00	30	3.2
5	111967266	10310053	Minzhu West Rd. Intersection	10:00	14:00	20	3.0
6	112015917	10305978	Donggang	10:00	18:00	30	3.3
7	111910421	10325543	Langongping	14:00	18:00	20	2.0
8	111897059	10366829	Anning District	10:00	18:00	20	2.3
9	111970885	10339850	Yanchang Rd. Intersection	11:00	15:00	20	2.1
10	111774002	10386268	Xigu District	8:00	12:00	30	3.5
11	112085409	10626368	Gaolan County	10:00	16:00	20	3.5
12	111827008	10864803	Lanzhou New District	8:00	14:00	20	4.2
13	112279239	10868423	Baiyin	8:00	18:00	30	6.6
14	112085409	10256862	Heping Village	10:00	15:00	20	2.4
15	112227743	10077823	Yuzhong County	14:00	18:00	20	3.1
16	112686557	9754533	Dingxi	8:00	18:00	30	6.4
17	111373082	9782756	Linxia	10:00	18:00	30	4.5
18	111693257	9665655	Guanghe County	13:00	18:00	20	3.0
19	111470477	10219942	Yongjing County	10:00	16:00	20	3.2
20	111051917	10670978	Honggu District	10:00	18:00	20	3.1
21	110627221	10825869	Haidong	10:00	18:00	30	4.0
22	110019508	11005135	Xi’ning	8:00	21:00	30	6.2
23	111458170	11081151	Yongdeng County	8:00	14:00	20	3.5
24	111332813	11400164	Tianzhu County	13:00	17:00	20	2.7
25	111117539	12000757	Gulang County	10:00	16:00	20	3.3
26	112233577	11655669	Jingtai County	10:00	16:00	20	3.0

**Table 5:** Hazardous materials optimal transportation plan based on the proposed method (plan A).

Distribution Center	Vehicle Type	Route	Loading/Capacity	Customer Name	Arrival−Departure Time	Delivery Volume (t)
F1	A	1	9.8/12.0	F1	6:34 AM	
				Yongdeng County	8:00 am−10:05 am	3.5
				Gulang County	11:32 am−1:31 pm	3.3
				Jingtai County	3:29 pm−5:19 pm	3
				F1	8:36 PM	
	C	2	17.1/20.0	F1	6:33 AM	
				Dingxi	8:00 am−10:38 am	6.4
				Railway Bureau of Lanzhou	12:08 pm−1:42 pm	3.2
				Guanghe County	2:58 pm−4:48 pm	3
				Linxia	5:27 pm−7:27 pm	4.5
				F1	9:33 PM	
	A	3	10.4/12.0	F1	9:17 AM	
				Gaolan County	10:00 am−12:05 pm	3.5
				Lanzhou New District	12:47 pm−3:13 pm	4.2
				Tianzhu County	4:51 pm−6:32 pm	2.7
				F1	8:26 PM	
	B	4	16.0/16.0	F1	6:55 AM	
				Baiyin	8:00 am−10:42 am	6.6
				Yanchang Road	11:42 am−1:05 pm	2.1
				Minzhu West Road	1:12 pm−3:02 pm	3
				Langongping	3:13 pm−4:33 pm	2
				Anning	4:44 pm−6:13 pm	2.3
				F1	6:38 PM	
	B	5	12.3/16.0	F1	10:02 AM	
				Yantan	10:05 am−11:45 am	3.5
				Heping Town	12:01 pm−1:33 pm	2.4
				Yuzhong County	2:00 pm−3:53 pm	3.1
				Donggang	4:36 pm−6:12 pm	3.3
				F1	6:21 PM	
F2	C	6	17.5/20.0	F2	5:11 AM	
				Xining	8:00 am−10:34 am	6.2
				Haidong	11:28 am−1:18 pm	4
				Honggu District	2:16 pm−4:09 pm	3.1
				Mogao Avenue	5:30 pm−6:44 pm	1.8
				Yintan	6:51 pm−8:23 pm	2.4
				F2	8:39 PM	
	A	7	6.7/12.0	F2	8:54 AM	
				Yongjing County	10:00 am−11:56 am	3.2
				Xigu	12:54 pm−2:34 pm	3.5
				F2	2:41 PM	

**Table 6:** Hazardous materials optimal transportation plan based on traditional method (plan B).

Distribution Center	Vehicle Type	Route	Loading/Capacity	Customer Name	Arrival–Departure Time	Delivery Volume (t)
F1	B	1	12.7/16.0	F1	7:46 am	
				Lanzhou Railway Bureau	8:00 am−9:34 am	3.2
				Linxia	11:26 am−1:26 pm	4.5
				Guanghe County	2:05 pm−3:55 pm	3
				Langongping	5:00 pm−6:20 pm	2
				F1	6:41 pm	
	C	2	17.3/20.0	F1	6:34 am	
				Dingxi	8:00 am−10:38 am	6.4
				Heping Village	11:47 am−1:19 pm	2.4
				Yanchang Rd. Intersection	1:37 pm−3:00 pm	2.1
				Donggang	3:11 pm−4:47 pm	3.3
				Yuzhong County	5:28 pm−7:21 pm	3.1
				F1	8:03 pm	
	B	3	13.1/16.0	F1	6:55 am	
				Baiyin	8:00 am−10:42 am	6.6
				Gaolan County	11:13 am−1:18 pm	3.5
				Minzhu West Rd. Intersection	2:00 pm−3:50 pm	3
				F1	4:02 pm	
	A	4	7.7/12.0	F1	7:00 am	
				Lanzhou New District	8:00 am−10:26 am	4.2
				Yantan	11:23 am−1:03 pm	3.5
				F1	1:05 pm	
F2	A	5	9.0/12.0	F2	6:43 am	
				Jingtai County	10:00 am−11:50 am	3
				Gulang County	1:48 pm−3:47 pm	3.3
				Tianzhu County	4:51 pm−6:32 pm	2.7
				F2	8:18 pm	
	C	6	17.5/20.0	F2	5:15 am	
				Xi’ning	8:00 am−10:34 am	6.2
				Haidong	11:26 am−1:16 pm	4
				Honggu	2:04 pm−3:57 pm	3.1
				Mogao Avenue	5:19 pm−6:33 pm	1.8
				Yintan	6:39 pm−8:11 pm	2.4
				F2	8:26 pm	
	B	7	12.5/16.0	F2	7:16 am	
				Xigu	7:22 am−9:02 am	3.5
				Yongjing County	10:00 am−11:56 am	3.2
				Yongdeng County	1:40 pm−3:45 pm	3.5
				An’ning	5:06 pm−6:35 pm	2.3
				F2	6:54 pm	

## Data Availability

The data used to support the findings of this study are available from the corresponding author upon request.
